# Independent prognostic impact of preoperative serum carcinoembryonic antigen and cancer antigen 15-3 levels for early breast cancer subtypes

**DOI:** 10.1186/s12957-018-1325-6

**Published:** 2018-02-12

**Authors:** Michiko Imamura, Takashi Morimoto, Takashi Nomura, Shintaro Michishita, Arisa Nishimukai, Tomoko Higuchi, Yukie Fujimoto, Yoshimasa Miyagawa, Ayako Kira, Keiko Murase, Kazuhiro Araki, Yuichi Takatsuka, Koshi Oh, Yoshikazu Masai, Kouhei Akazawa, Yasuo Miyoshi

**Affiliations:** 1 Department of Surgery, Division of Breast and Endocrine Surgery, Hyogo College of Medicine, Mukogawa-cho 1-1, Nishinomiya City, Hyogo 663-8501 Japan; 2 Department of Breast Surgery, Yao Municipal Hospital, Ryuka-cho 1-3-1, Yao City, Osaka 581-0069 Japan; 3 Department of Surgery, Kobe Adventist Hospital, Arinodai,Kita-ku 8-4-1, Kobe, Hyogo 651-1312 Japan; 4 Masai Breast Clinic, Funado-cho 2-1-205, Ashiya, Hyogo 659-0093 Japan; 5 Department of Medical Informatics, Niigata University Medical & Dental Hospital, Chuo-ku, Niigata Japan

**Keywords:** Breast cancer, Tumor marker, CEA, CA15-3, Prognosis

## Abstract

**Background:**

Although the prognosis for operable breast cancers is reportedly worse if serum carcinoembryonic antigen (CEA) and cancer antigen 15-3 (CA15-3) levels are above normal, the usefulness of this prognosis is limited due to the low sensitivity and specificity; in addition, the optimal cutoff levels remain unknown.

**Methods:**

A total of 1076 patients who were operated for breast cancers (test set = 608, validation set = 468) without evidence of metastasis were recruited, and their baseline and postoperative serum CEA and CA15-3 levels were analyzed. The optimal cutoff values of CEA and CA15-3 for disease-free survival (DFS) were 3.2 ng/mL and 13.3 U/mL, respectively, based on receiver operating characteristic curve and area under the curve analyses.

**Results:**

The DFS of patients with high CEA levels (CEA-high: *n* = 191, 5-year DFS 70.6%) was significantly worse (*p* < 0.0001) than that of CEA-low patients (*n* = 885, 5-year DFS 87.2%). There was a significant difference in DFS (*p* < 0.0001) between CA15-3-high and CA15-3-low patients (*n* = 314 and *n* = 762, respectively; 5-year DFS 71.8 vs. 89.3%). Significant associations between DFS and CA15-3 levels were observed irrespective of the subtypes. Multivariable analysis indicated that tumor size, lymph node metastasis, tumor grade, and CEA (*p* = 0.0474) and CA15-3 (*p* < 0.0001) levels were independent prognostic factors (hazard ratio [HR] 1.520, 95% confidence interval [CI] 1.005–2.245 for CEA; HR 2.088, 95% CI 1.457–2.901 for CA15-3).

**Conclusions:**

These findings suggest that CEA and CA15-3 levels might be useful for predicting the prognosis of patients with operable early breast cancer irrespective of the subtype. Serum levels at baseline may reflect tumor characteristics for metastatic potential even when these levels are within the normal ranges.

**Electronic supplementary material:**

The online version of this article (10.1186/s12957-018-1325-6) contains supplementary material, which is available to authorized users.

## Background

Serum tumor markers for breast cancers, including carcinoembryonic antigen (CEA) and cancer antigen 15-3 (CA15-3), are widely used in daily clinical practice. Serial measurements of these markers are useful for detecting or monitoring treatment efficacy in metastatic breast cancer patients because the levels of these markers are generally elevated above the normal range in these patients [1, 2]. In addition to their value for diagnostic and monitoring purposes, elevated levels of CEA and CA15-3 at the time of systemic recurrence of breast cancer are significantly associated with patient prognosis [3, 4]. Although serial postoperative measurement of these markers for detecting recurrence and monitoring treatment is recommended by the European Group on Tumor Markers [5], their utility for the screening and diagnosis of early breast cancers without metastasis is limited due to the low sensitivity and specificity of these markers.

Even among patients without metastasis at diagnosis of breast cancer, serum levels of CEA and CA15-3 reportedly exceed the normal range in 7.2–32% and 5.5–20% of patients, respectively [6–13]. Interestingly, in these studies, the prognosis of patients with elevated preoperative levels of CEA and CA15-3 was significantly worse than that of patients with normal levels. For example, the 5-year disease-free survival (DFS) of patients with elevated CEA (77.9 vs. 57.4%; *p* = 0.004) and CA15-3 (88.8 vs. 74.4%; *p* = 0.002) was significantly worse than that of patients with normal levels [13]. Similarly, the overall survival (OS) was significantly worse for patients with elevated CEA (88.8 vs. 77.4%; *p* = 0.002) as well as elevated CA15-3 (90.8 vs. 71.7%; *p* < 0.001) than for those with values in the normal ranges. In addition, multivariable analyses showed that serum levels of preoperative CEA and CA15-3 were independent prognostic factors for both DFS and OS [8, 11, 13]. Although the clinical usefulness of elevated CEA and CA15-3 levels for predicting patient prognosis in early breast cancers has been established, these patients usually accounted for a small proportion of the population, thus emphasizing the need for more sensitive markers [14].

In order to distinguish breast cancer from non-cancerous patients, the cutoff values of CEA and CA15-3 are usually defined on the basis of the 95% percentile of healthy individuals and the upper limits of these markers are used as the cutoff values in analytical studies of the prognostic significance of early breast cancers. Considering that serum levels of these tumor markers in operable breast cancer patients are significantly higher than those in healthy individuals even though they are within the normal range [15–17], preoperative marker levels, even without being elevated beyond the normal range, may be meaningful. Nevertheless, less attention has been paid to the prognosis of patients with normal levels of CEA and CA15-3 at baseline, and the optimal cutoff values remain to be elucidated. Furthermore, the postoperative significance of these markers has hardly been studied.

To evaluate the prognostic significance of preoperative tumor marker levels in early breast cancers, we investigated the baseline and postoperative serum levels of CEA and CA15-3. In addition, breast cancer subtypes were considered in order to analyze the prognostic significance of these markers.

## Methods

### Patient eligibility

Breast cancer patients who underwent operations at Hyogo College of Medicine Hospital between February 2005 and December 2014 and at Yao Municipal Hospital between May 2004 and December 2010 were consecutively recruited for this retrospective study. We excluded patients with non-invasive carcinomas, concurrent bilateral breast cancers, and male breast cancers. Finally, we obtained data on serum CEA and CA15-3 levels at baseline for 608 patients from Hyogo College of Medicine Hospital (test set) and 468 from Yao Municipal Hospital (validation set); thus, 1076 operated patients were eligible for this study. All patients were histologically confirmed to have invasive breast cancers, which were diagnosed as non-metastatic via imaging.

### Adjuvant treatments and patient follow-up

A total of 512 patients underwent chemotherapy preoperatively (*n* = 185), postoperatively (*n* = 301), and both (*n* = 26). Anthracycline-containing regimens, sequential administration of anthracycline and taxanes, taxane-based regimens, and unspecified regimens were administered to 100, 248, 140, and 21 patients, respectively (the details of the chemotherapy regimen for three patients were not known). A total of 804 patients were treated with endocrine therapies, including luteinizing hormone-releasing hormone plus tamoxifen (*n* = 166), luteinizing hormone-releasing hormone plus aromatase inhibitor (*n* = 2), tamoxifen (*n* = 128), and aromatase inhibitors (*n* = 489). Endocrine therapies for the remaining 19 patients were switched from luteinizing hormone-releasing hormone plus tamoxifen (*n* = 5) or tamoxifen (*n* = 14) to aromatase inhibitors. The 30 patients with estrogen receptor (ER)-positive breast cancers did not receive any endocrine therapy, and therapy for 35 patients was unknown. For 94 patients, endocrine therapy was administered both pre- and post-operatively. Administration of adjuvant treatments was determined based on the current St Gallen guidelines at that time [18–22].

The majority of the patients visited the hospital postoperatively every 3 to 6 months for 3 years, and every 6 to 12 months thereafter; at which time, blood tests, physical examinations, and mammography were performed annually. The median follow-up time was 46.8 months (range 1–127 months). During the follow-up, 135 patients showed recurrence in the locoregional and lymph nodes (*n* = 45), ipsilateral or contralateral breast (*n* = 15), bone (*n* = 37), lungs (*n* = 26), liver (*n* = 14), pleura (*n* = 5), brain (*n* = 10), and others (*n* = 2). The DFS was defined as the time from operation to the first recurrence or death due to any reasons (*n* = 17).

### Serum CEA and CA15-3 examination

Baseline CEA and CA15-3 (*n* = 1076) data were obtained preoperatively except for patients treated with neoadjuvant therapies, whose samples were collected before treatment. We collected postoperative data for CEA (*n* = 861) and CA15-3 (*n* = 858) from patients who had no recurrence at 6 to 12 months after the operation. From patients at the Hyogo College of Medicine Hospital, data from serial measurements obtained at 1, 3, 6, and 12 months postoperatively were available.

Serum levels of CEA and CA15-3 were determined by electrochemiluminescence immunoassay (ECLIA) and chemiluminescence enzyme immunoassay (CLEIA), respectively, at Hyogo College of Medicine Hospital using a kit provided by Roche Diagnostics Ltd. (Rotkreuz, Switzerland) for CEA and by Fujirebio Inc. (Tokyo, Japan) for CA15-3. For CEA, each sample was incubated for 9 min at 37 °C with biotinylated anti-CEA antibody and ruthenylated anti-CEA antibody (Ru (bpy)_3_-anti-CEA antibody), after which streptavidin-coated magnetic microparticles (SA magnet MP) were mixed in and incubated for 9 min at 37 °C. Next, a separate unbound Ru (bpy)_3_-anti-CEA antibody from the reaction mixture and CEA levels were determined by calibrating emission intensity generated by the bound Ru (bpy)_3_-anti-CEA antibody to the SA magnet MP using a photomultiplier tube [23]. In order to detect CA15-3 levels, each sample and anti-CA15-3 antibody-bound particles were mixed and incubated for 8 min at 37 °C. After the collection of these particles, which had been incubated for 8 min at 37 °C with an alkaline phosphatase-labeled anti-CA15-3 antibody, particles bound to the immune complex were separated and incubated for 4 min at 37 °C with substrate. CA15-3 levels then were determined by measuring the emission intensity catalyzed by alkaline phosphatase [24].

At Yao Municipal Hospital, CEA and CA15-3 were measured with a chemiluminescent immunoassay (CLIA) using a kit (ADVIA Centaur), provided by Siemens Healthineers Japan (Tokyo, Japan). Briefly, each sample was incubated with an acridinium ester-labeled antibody at 37 °C for 7.5 min in water and 9.7 min in a wash solution. After the bound and free complexes had been separated, they were mixed with oxidant and the CEA and CA15-3 levels were measured [25].

### Statistical analysis

The relationships between the clinicopathological characteristics and serum levels of CEA or CA15-3 were determined by Chi-square, Fisher’s exact, or Mann-Whitney tests, as appropriate. Differences in DFS in Kaplan-Meier plots were compared by means of log-rank tests. Univariable and multivariable analyses of the risk factors for DFS were performed with a Cox proportional hazards model to obtain hazard ratios (HRs) and 95% confidence intervals (95% CIs). Postoperative changes in CEA and CA15-3 levels were statistically analyzed using Wilcoxon signed-ranks tests. The statistical significance was set at *p* < 0.05 except for multiple comparisons of the postoperative course adjusted with Bonferroni correction, for which the significance was set at *p* < 0.005. All statistical calculations were performed using JMP Pro 11 (SAS Institute Inc., Cary, NC, USA).

## Results

### Determination of the baseline CEA and CA15-3 cutoff values for DFS

**Fig. 1:**
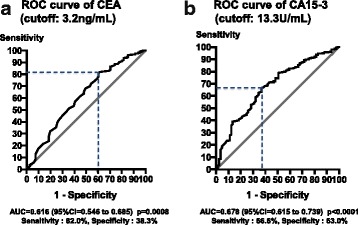
Receiver operating characteristics (ROC) curves of CEA (**a**) and CA15-3 (**b**) levels for disease-free survival. The cutoff values were set at 3.2 ng/mL for CEA and 13.3 U/mL for CA15-3 based on the areas under the curve (AUC), which were 0.616 (95% confidence interval [CI] 0.546–0.685, *p* = 0.0008) for CEA and 0.678 (95% CI 0.615–0.739, *p* < 0.0001) for CA15-3

The cutoff values of CEA and CA15-3 for DFS were determined based on the analysis of receiver operating characteristics (ROC) curves calculated using the Youden index for areas under the curve (AUC). Using the data obtained at Hyogo College of Medicine (test set), the cutoff values for CEA and CA15-3 were set at 3.2 ng/mL (AUC 0.616, 95% CI 0.546–0.685, *p* = 0.0008) and 13.3 U/mL (AUC 0.678, 95% CI 0.615–0.739, *p* < 0.0001), respectively (Fig. 1). The sensitivity and specificity were 82.0 and 38.3% for CEA and 66.6 and 63.0% for CA15-3, respectively.

**Fig. 2:**
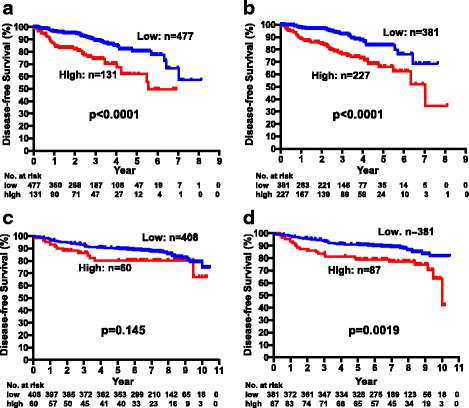
Disease-free survival of patients from Hyogo College of Medicine with high (*n* = 131) and low (*n* = 477) CEA levels (**a**) and high (*n* = 227) and low (*n* = 381) CA15-3 levels (**b**). Disease-free survival of patients from Yao Municipal Hospital with high (*n* = 60) and low (*n* = 408) CEA levels (**c**) and high (*n* = 87) and low (*n* = 381) CA15-3 levels (**d**)

Using these cutoff values, we classified the 608 patients from Hyogo College of Medicine as CEA high (*n* = 131) or CEA low (*n* = 477) and as CA15-3 high (*n* = 227) or CA15-3 low (*n* = 381). As shown in Fig. 2a, the DFS of patients who were serum CEA-high at baseline (5-year DFS 61.9%) was significantly worse than the DFS of those who were CEA-low (5-year DFS 80.3%, *p* < 0.0001). Similarly, there was a significant association between serum CA15-3 levels at baseline and DFS (5-year DFS 66.0 vs 83.3%, *p* < 0.0001; Fig. 2b). To validate these CEA and CA15-3 cutoff values, we examined the DFS of 468 patients treated at Yao Municipal Hospital. Although the difference was not statistically significant (*p* = 0.145, Fig. 2c), the DFS of the CEA-high group (5-year DFS 80.4%) was worse than that in the CEA-low group (5-year DFS 89.9%).There was a significant association between CA15-3 levels and DFS (5-year DFS 90.9% for low and 79.0% for high, *p* = 0.0019, Fig. 2d). Since we confirmed the usefulness of these cutoff values in the validation set, further analyses were done using combined data from Hyogo College of Medicine and Yao Municipal Hospital. The Kaplan-Meier plots of all patients according to CEA levels (5-year DFS of CEA-high 70.6%, *n* = 191 vs. CEA-low 87.2%, *n* = 885, *p* < 0.0001) and CA15-3 levels (5-year DFS of CA15–3-high: 71.8%, *n* = 314 vs. CA15-3-low: 89.3%, *n* = 762, *p* < 0.0001) are shown in Additional file 1: Figure S1.

### DFS in relation to serum CEA and CA15-3 levels at baseline according to subtypes

**Fig. 3:**
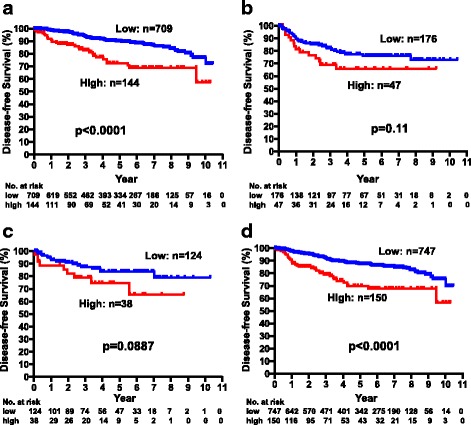
Disease-free survival of patients with high and low CEA levels in ER-positive (**a**), ER-negative (**b**), HER2-positive, (**c**) and HER2-negative (**d**) breast cancers

**Fig. 4:**
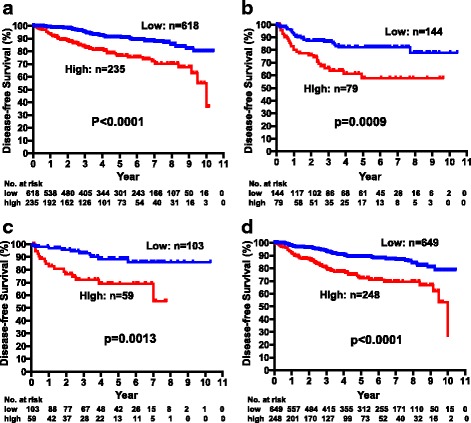
Disease-free survival of patients with high and low CA15-3 levels in ER-positive (**a**), ER-negative (**b**), HER2-positive, (**c**) and HER2-negative (**d**) breast cancers

The DFS of all patients were further analyzed according to ER or human epidermal growth factor receptor 2 (HER2) status in relation to CEA and CA15-3 levels. The DFS of patients in the CEA-high group was significantly worse than that of patients in the CEA-low group among patients with ER-positive breast cancers (*p* < 0.0001), but there was no significant association in patients with ER-negative breast cancers (*p* = 0.11) (Fig. 3a, b). Significant (*p* < 0.0001) and marginally significant (*p* = 0.0887) associations between CEA level and DFS were observed in HER2-negative and HER2-positive breast cancers, respectively (Fig. 3c, d). Significant associations between DFS and CA15-3 levels were consistently observed in both ER-positive (*p* < 0.0001) and ER-negative (*p* = 0.0009) as well as HER2-positive (*p* = 0.0013) and HER2-negative (*p* < 0.0001) patients (Fig. 4a–d).

### Relationship between clinicopathological characteristics and serum CEA and CA15-3 levels at baseline

Table 1 shows the relationships between the levels of these tumor markers at baseline and the clinicopathological factors. In the CEA-high subset, significantly higher frequencies were observed for postmenopausal status (*p* < 0.0001), larger tumor size (*p* < 0.0001), lymph node metastasis (*p* = 0.036), progesterone receptor (PgR) negativity (*p* = 0.005), and HER2 positivity (*p* = 0.044). Similarly, in the CA15-3-high subset, the frequencies of larger tumor size (*p* < 0.0001), lymph node metastasis (*p* < 0.0001), ER negativity (*p* = 0.021), PgR negativity (*p* = 0.013), HER2 positivity (*p* = 0.024), and chemotherapy administration (*p* = 0.0011) were significantly higher.

**Table 1:** Clinicopathological characteristics of breast cancers according to CEA or CA15–3 levels

Characteristics	CEA high^a^ (*n* = 191)	CEA low^a^ (*n* = 885)	*p* value	CA15-3 high^b^ (*n* = 314)	CA15-3 low^b^ (*n* = 762)	*p* value
Menopausal status						
Pre-	30 (8.3)^c^	330 (91.7)	< 0.0001	96 (26.7)	264 (73.3)	0.200
Post-	157 (22.3)	546 (77.7)		214 (30.4)	489 (69.6)	
Unknown	4 (30.8)	9 (69.2)		4 (30.8)	9 (69.2)	
Histological type						
IDC	165 (16.9)	806 (83.1)	0.096	278 (28.6)	693 (71.4)	0.276
ILC	8 (20.5)	31 (79.5)		11 (28.2)	28 (71.8)	
Others	18 (27.3)	48 (72.7)		25 (37.9)	41 (62.1)	
Tumor size						
≤ 2 cm	87 (13.6)	552 (86.4)	< 0.0001	157 (24.6)	482 (75.4)	< 0.0001
> 2 cm	102 (23.9)	324 (76.1)		155 (36.4)	271 (63.6)	
Unknown	2 (18.2)	9 (81.8)		2 (18.2)	9 (81.8)	
Lymph node metastasis						
Negative	121 (16.3)	622 (83.7)	0.036	186 (25.0)	557 (75.0)	< 0.0001
Positive	64 (20.2)	253 (79.8)		123 (38.8)	194 (61.2)	
Not examined	6 (37.5)	10 (62.5)		5 (31.3)	11 (68.8)	
Tumor grade						
1	90 (16.0)	471 (84.0)	0.251	156 (27.8)	405 (72.2)	0.424
2 + 3	90 (18.8)	389 (81.2)		144 (30.1)	335 (69.9	
Unknown	11 (30.6)	25 (69.4)		14 (38.9)	22 (61.1)	
Estrogen receptor						
Positive	144 (16.9)	709 (83.1)	0.168	235 (27.6)	618 (72.5)	0.021
Negative	47 (21.1)	176 (78.9)		79 (35.4)	144 (64.6)	
Progesterone receptor						
Positive	113 (15.4)	622 (84.6)	0.0025	197 (26.8)	538 (73.2)	0.013
Negative	78 (23.2)	258 (76.8)		115 (34.2)	221 (65.8)	
Unknown	0 (0.0)	5 (100.0)		2 (40.0)	3 (60.0)	
HER2 status						
Negative	150 (16.7)	747 (83.3)	0.044	248 (27.6)	649 (72.4)	0.024
Positive	38 (23.5)	124 (76.5)		59 (36.4)	103 (63.6)	
Unknown	3 (17.6)	14 (82.4)		7 (41.2)	10 (58.8)	
Ki67 status^d^						
Low	65 (20.2)	256 (79.8)	0.764	99 (30.8)	222 (69.2)	0.098
High	60 (21.3)	222 (78.7)		105 (37.2)	177 (62.8)	
Unknown	66 (14.0)	407 (86.0)		110 (23.3)	365 (76.7)	
Chemotherapy						
Yes	89 (17.4)	423 (82.6)	0.870	171 (33.4)	341 (66.6)	0.0011
No	92 (18.0)	420 (82.0)		123 (24.0)	389 (76.0)	
Unknown	10 (19.2)	42 (80.8)		20 (38.5)	32 (61.5)	

*IDC* invasive ductal carcinoma, *ILC* invasive lobular carcinoma

^a^High: ≥ 3.2ng/mL, low: < 3.2ng/mL

^b^High ≥ 13.3 U/mL, low < 13.3 U/mL

^c^(%)

^d^Low < 20%, high ≥ 20%

### Correlations of first metastatic sites with preoperative serum CEA or CA15-3 levels

The correlations between baseline CEA or CA15-3 levels and first metastatic sites were analyzed, as shown in Table 2. The proportion of patients in the CA15-3-high group was significantly higher than that in the bone metastasis (*p* < 0.0001) and the proportions of patients in the CEA-high and CA15-3-high groups were significantly higher than that in the visceral metastasis (*p* = 0.0006 for CEA and *p* < 0.0001 for CA15-3). On the other hand, there were no significant associations between the frequencies of metastases to locoregional or soft tissue and CEA or CA15-3 levels.

**Table 2:** Correlation between serum CEA or CA15-3 levels at baseline and first metastatic sites

Metastatic sites	CEA high (*n* = 191)	CEA low^a^ (*n* = 885)	*p* value	CA15-3 high (*n* = 314)	CA15-3 low^b^ (*n* = 762)	*p* value
No metastasis						
	154 (16.4)^c^	787 (83.6)		249 (36.0)	692 (64.0)	
Visceral metastasis						
	19 (36.5)	33 (63.5)	0.0006	31 (59.6)	21 (40.4)	< 0.0001
Bone^d^						
	7 (25.0)	21 (75.0)	0.298	14 (50.0)	14 (50.0)	< 0.0001
Locoreginal and soft tissue^d^						
	11 (20.0)	44 (80.0)	0.458	20 (36.4)	35 (63.4)	0.119

^a^High ≥ 3.2 ng/mL, low: < 3.2 ng/mL

^b^High ≥ 13.3 U/mL, low < 13.3 U/mL

^c^(%)

^d^Without visceral metastasis

### Univariable and multivariable analyses of DFS including baseline serum CEA and CA15-3 levels

Univariable analysis showed that tumor size, lymph node metastasis, tumor grade, ER status, PgR status, chemotherapy administration, CEA levels, and CA15-3 levels were significant prognostic factors for DFS (Table 3). Multivariable analysis including these factors revealed that tumor size (*p* < 0.0001), lymph node metastasis (*p* < 0.0001), tumor grade (*p* = 0.014), ER status (*p* = 0.024), chemotherapy administration (*p* = 0.0021), and CEA (*p* = 0.047) and CA15-3 (*p* < 0.0001) levels were independent prognostic factors (HR 1.520, 95% CI 1.005–2.245 for CEA high; HR 2.088, 95% CI 1.457–2.901 for CA15-3 high) (Table 3).

**Table 3:** Univariable and multivariable analyses of relapse-free survival

	*n*	Univariable analysis HR (95% CI)^a^	*p* value	Multivariable analysis HR (95% CI)^a^	*p* value
Menopausal status					
Pre-	360	1.365 (0.958–1.985)	0.086		
Post-	703	1.00			
Tumor size					
≤ 2.0 cm	639	1.00	< 0.0001	1.00	< 0.0001
> 2 cm	426	2.964 (2.135–4.157)		2.410 (1.645–3.575)	
Lymph node metastasis					
Negative	743	1.00	< 0.0001	1.00	< 0.0001
Positive	317	2.990 (2.160–4.154)		2.568 (1.742–3.800)	
Tumor grade					
1	561	1.00	0.0003	1.00	0.014
2+3	479	1.859 (1.331–2.624)		1.627 (1.101–2.420)	
Estrogen receptor status					
Positive	853	1.00	< 0.0001	1.00	0.024
Negative	223	2.089 (1.471–2.925)		1.964 (1.094–3.610)	
Progesterone receptor status					
Positive	735	1.00	< 0.0001	1.00	0.843
Negative	336	1.946 (1.406–2.681)		1.054 (0.614–1.736)	
HER2 status					
Negative	897	1.00		0.186	
Positive	162	1.345 (0.861–2.022)			
Chemotherapy					
No	512	1.00	0.0221	1.00	0.0021
Yes	512	1.480 (1.107–2.201)		0.509 (0.335–0.780)	
CEA level^b^					
Low	885	1.00	< 0.0001	1.00	0.047
High	191	2.415 (1.057–2.091)		1.520 (1.005–2.245)	
CA15-3 level^c^					
Low	762	1.00	< 0.0001	1.00	< 0.0001
High	314	2.824 (2.050–3.887)		2.088 (1.457–2.901)	

^a^Hazard ratio (95% confidence interval)

^b^High: ≥ 3.2ng/mL, low < 3.2 ng/mL

^c^High: ≥ 13.3 U/mL, low < 13.3 U/mL

### Changes in serum CEA and CA15-3 levels during postoperative course and DFS in relation to postoperative serum CEA and CA15-3 levels

**Fig. 5:**
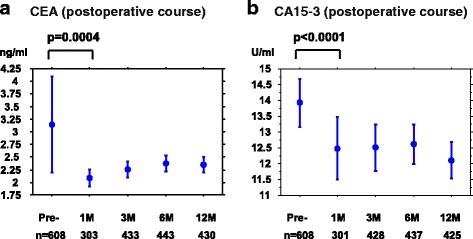
Changes in CEA (**a**) and CA15-3 (**b**) levels at baseline and 1, 3, 6, and 12 months postoperatively (mean and 95% confidence interval)

Serial data of the levels of tumor markers during the postoperative course obtained 1, 3, 6, and 12 months postoperatively were available for 608 patients with breast cancers at Hyogo College of Medicine Hospital. The mean (3.15 ng/mL) and 95% CI (2.20–4.10 ng/mL) of CEA levels at baseline decreased significantly 1 month postoperatively (mean 2.09 ng/mL, 95% CI 1.92–2.26 ng/mL; *p* = 0.0004) (Fig. 5a). Similarly, CA15-3 levels at baseline (mean 13.78 U/mL, 95% CI 13.10–14.47 U/mL) had significantly decreased at 1 month postoperatively (mean 12.48 U/mL, 95% CI 11.49–13.47 U/mL; *p* < 0.0001) (Fig. 5b).

**Fig. 6:**
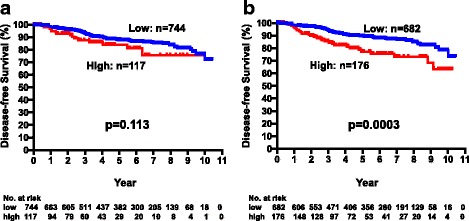
Disease-free survival of patients with high (*n* = 117) and low (*n* = 744) postoperative CEA levels (**a**) and high (*n* = 176) and low (*n* = 682) CA15-3 levels (**b**)

Next, we analyzed the DFS in relation to the postoperative CEA and CA15-3 levels (between 6 to 12 months postoperatively), as shown in Fig. 6. DFS did not differ significantly between the postoperative CEA-high and CEA-low subsets (*p* = 0.113). There was a significant difference between postoperative CA15-3-high and CA15-3-low subsets (*p* = 0.0003, Fig. 6b), but the difference was smaller than for those levels at baseline (Additional file 1: Figure S1).

## Discussion

The results of the present study verified that the DFS was significantly worse in 1076 operated breast cancer patients in the serum CEA-high group at baseline than for those in the serum CEA-low group. There was a similarly significant association between DFS and serum CA15-3 levels at baseline. These significant associations appear to be consistently observed irrespective of ER or HER2 status. Since CEA and CA15-3 levels at baseline were significant prognostic factors independent of other clinicopathological characteristics, combining the two markers is useful for the identification of patients with poor prognosis.

A number of studies have established that prognoses are worse when CEA and CA15-3 levels elevated above the normal upper range are used as cutoff values [6–8, 10, 11, 13]. Sandri et al. assessed prognosis based on CA15-3 levels for groups divided by quartile values and reported a significant association with distant metastases and death in the group with the highest quartile (> 20 U/mL) in comparison with that of the other groups (≤ 20 U/mL) [26]. Li et al. used the mean value of CA15-3 as a cutoff for the prognosis of breast cancer patients [27]. Similar to our study, Samy et al. determined the best cutoff values (4.55 ng/mL for CEA and 26.5 U/mL for CA15-3) based on maximization of the sum of the sensitivity and specificity by ROC curve analysis [28]; however, these cutoff values were obtained for only 89 patients and with a short follow-up (up to 18 months). To the best of our knowledge, our study is the first to determine the optimal cutoff values based on ROC curves in a large cohort (*n* = 1076) with a longer follow-up (median 46.8 months). On the basis of our cutoff values, CEA-high and CA15-3-high patients accounted for 17.8 and 29.2% of all patients, respectively. The ratio of CEA-high patients in our study (17.8%) was similar to that reported previously (23.8%) [28]. The ratio of patients with high levels of CA15-3 in our study (29.2%) was also similar to that reported by Samy et al. (35%), in which the cutoff value was determined by ROC curve analysis [28] and to that for the highest quartile in Sandri et al.’s study (23.6%) [26].

Li et al. reported that preoperative CA15-3 levels were significantly associated with worse prognosis only for luminal A breast cancers [27]. In addition, Shao et al. demonstrated the prognostic significance of elevated CEA levels in luminal B breast cancers [13]. In contrast to Li et al.’s findings, our data suggest the prognostic significance of CEA and CA15-3 levels regardless of ER or HER2 status. The reason for the discrepancy in our results is not known, but one partial explanation may be the smaller sample size (*n* = 368) and different cutoff points (mean values) used by Li et al. Interestingly, we observed no significant associations between the baseline levels of CEA or CA15-3 and the frequency of first metastasis in locoregional or soft tissue (Table 2). Consistent with our observation, CA15-3 has been shown to be a significant prognostic factor for distant metastasis (*p* < 0.01), but not for locoregional events (*p* = 0.45) [26]. No significant association between metastatic sites and either elevated CEA or CA15-3 has been reported for metastatic breast cancers [29]. However, Tampellini et al. reported that patients with elevated CA15-3 levels frequently had metastasis in the liver (72%) and less frequently in the skin and lymph nodes (28%) [30]. Consistent with these findings, among patients with elevated CA15-3 level, metastasis was significantly increased in the bone (61%) and the liver (65%), but not in the soft tissue (50%), and elevated CEA levels were associated with metastasis in the liver (48%), but not the soft tissue (26%) [3]. Although the correlation between tumor marker levels and metastatic sites is not yet conclusive, elevated levels of these markers appear to be linked to the bone and visceral but not with the soft tissue metastases. Since CEA and CA15-3 are cell surface glycoproteins expressed in cancer cells and released into the bloodstream [14], elevated levels of tumor markers may reflect the efflux of these glycoproteins into the bloodstream.

A significant decrease in postoperative compared to preoperative serum CEA and CA15-3 levels has been reported [6, 28]. We also found that the reduction of these markers occurred within 1 month postoperatively. The fact that these reductions follow a resection clearly means that an increase in the levels of these markers is generated from primary breast cancers and not from micrometastases. DFS and OS were significantly worse for patients whose CEA levels decreased more than 33% postoperatively [6]. A possible hypothesis is that a larger reduction in the two markers reflects a higher production of these markers in primary breast cancers, resulting in worse prognosis. In line with this hypothesis, our study found the worse DFS in patients with high serum levels of the two markers at baseline and low postoperative levels (data not shown). The prognostic significance of these markers at baseline is thus more prominent than their postoperative course. As mentioned above, in a majority of cases, increases in serum levels of these markers seem to be generated from primary breast cancers rather than from micrometastases. The fact that the prognosis is worse even for patients whose marker levels decrease postoperatively strongly indicates the significance of baseline rather than postoperative levels as prognostic indicators. Although CEA and CA15-3 were significantly associated with tumor size, the associations between high marker levels and worse prognosis were established independently by multivariable analyses. CA15-3 (MUC1) is a glycoprotein member of the mucin family, and MUC1 has been well established as playing a significant role in metastasis by promoting cell migration and activation of signaling pathways including Src [31]. These observations suggest that not only large tumor burden but also metastatic potential of breast cancers are associated with high levels of CEA and CA15-3. The limitation of the current study is that we determined the cutoff values based on test set and applied these values to the validation set, in which CEA and CA15-3 were measured using different methods; thus, the optimal cutoff values have yet to be determined. Further validation of these findings, including the optimal cutoff values using large sample sizes, is needed.

## Conclusions

In conclusion, the results of the present study demonstrated that the outcomes for patients with high levels of CEA and CA15-3 at baseline rather than postoperatively are significantly associated with worse DFS than for those with correspondingly low levels of the two markers. In addition, the prognostic significance of these markers was independent of tumor size, lymph node metastasis, or tumor grade and irrespective of ER or HER2 status. Since examination of these markers is widely used in daily clinical practice, the data obtained in the present study yielded important information for identifying patients with poor prognosis and selecting candidates for adjuvant treatments.

## Additional file

Additional file 1: **Figure S1.** Disease-free survival of all patients with high (*n*=191) and low (*n*=885) CEA levels (a) and high (*n*=314) and low (*n*=762) CA15-3 levels (b). (PPTX 117 kb)
