# Supplementary material for: Independent prognostic impact of preoperative serum carcinoembryonic antigen and cancer antigen 15-3 levels for early breast cancer subtypes
Source: World J Surg Oncol. 2018 Feb 12;16:26. doi: 10.1186/s12957-018-1325-6 (PMC5809836; doi:10.1186/s12957-018-1325-6)
Supplement: Supplementary file 1 — Figure S1. Disease-free survival of all patients with high (n=191) and low (n=885) CEA levels (a) and high (n=314) and low (n=762) CA15-3 levels (b). (PPTX 117 kb) [file 12957_2018_1325_MOESM1_ESM.pptx]

## Slide 1
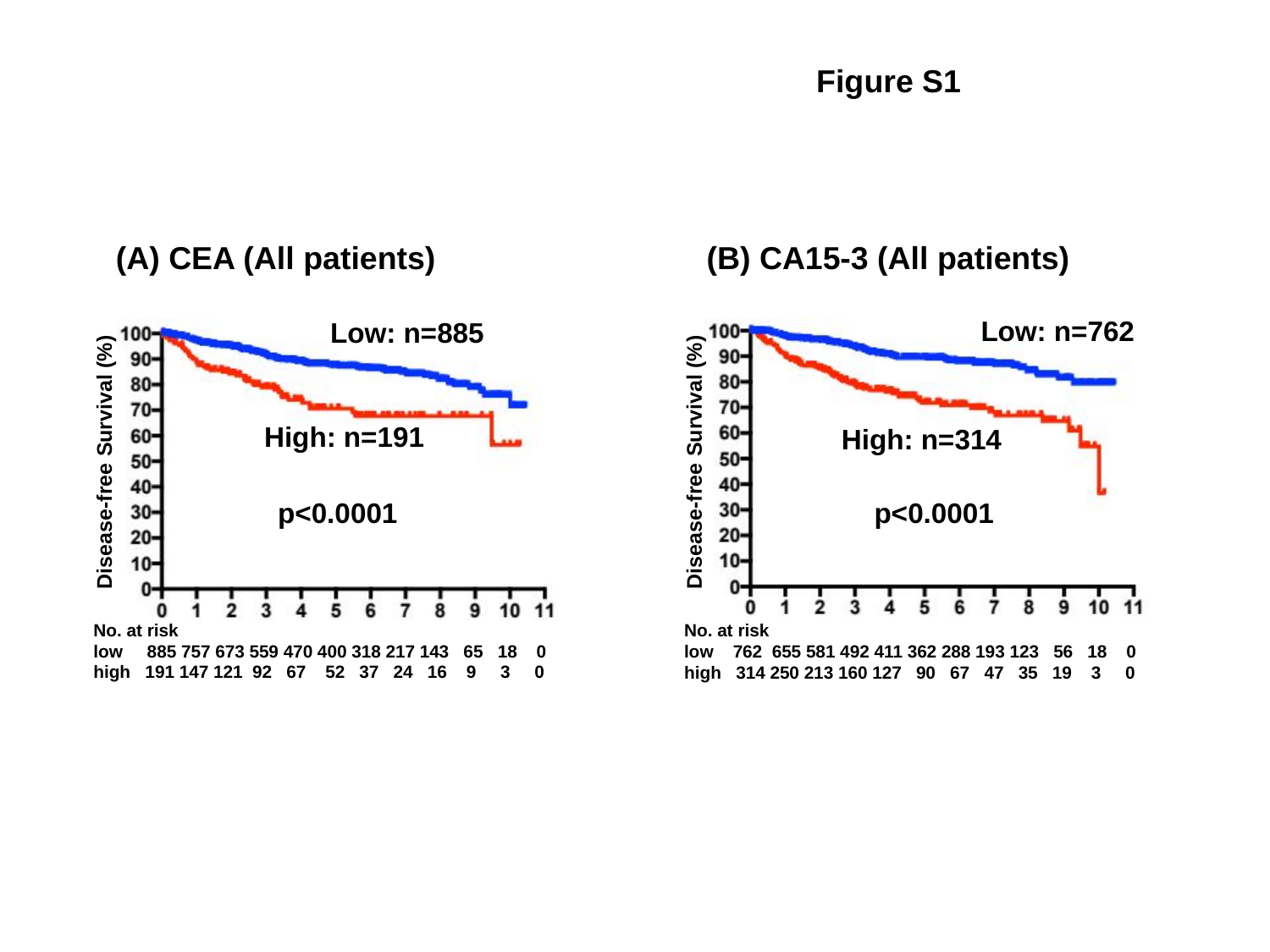

Figure S1
(A) CEA (All patients)
(B) CA15-3 (All patients)
Low: n=762
Low: n=885
High: n=191
High: n=314
Disease-free Survival (%)
Disease-free Survival (%)
p<0.0001
p<0.0001
No. at risk
low 885 757 673 559 470 400 318 217 143 65 18 0
high 191 147 121 92 67 52 37 24 16 9 3 0
No. at risk
low 762 655 581 492 411 362 288 193 123 56 18 0
high 314 250 213 160 127 90 67 47 35 19 3 0
